# Supplementary material for: Isorhamnetin Suppresses Human Gastric Cancer Cell Proliferation through Mitochondria-Dependent Apoptosis
Source: Molecules. 2022 Aug 15;27(16):5191. doi: 10.3390/molecules27165191 (PMC9415531; doi:10.3390/molecules27165191)
Supplement: Supplementary file 1 [file molecules-27-05191-s001.zip › molecules-1873404-supplementary.pdf]

Supplementary Table S1
Primer list

primer	sequence
scrambled-F	CCGGGCGCGATAGCGCTAATAATTTCTCGAGAAATTATTAGCGCTATCGCGCTTTTTG
scrambled-R	AATTCAAAAAGCGCGATAGCGCTAATAATTTCTCGAGAAATTATTAGCGCTATCGCGC
shBax-1F	CCGGGCCCACCAGCTCTGAGCAGATCTCGAGATCTGCTCAGAGCTGGTGGGCTTTTTG
shBax-1R	AATTCAAAAAGCCCACCAGCTCTGAGCAGATCTCGAGATCTGCTCAGAGCTGGTGGGC
shBax-2F	CCGGATCATCAGATGTGGTCTATAACTCGAGTTATAGACCACATCTGATGATTTTTTG
shBax-2R	AATTCAAAAAATCATCAGATGTGGTCTATAACTCGAGTTATAGACCACATCTGATGAT
gapdh-qF	GGCATCCTGGGCTACACTGA
gapdh-qR	GAGTGGGTGTCGCTGTTGAA
Bax-qF	CCCGAGAGGTCTTTTTCCGAG
Bax-qR	CCAGCCCATGATGGTTCTGAT

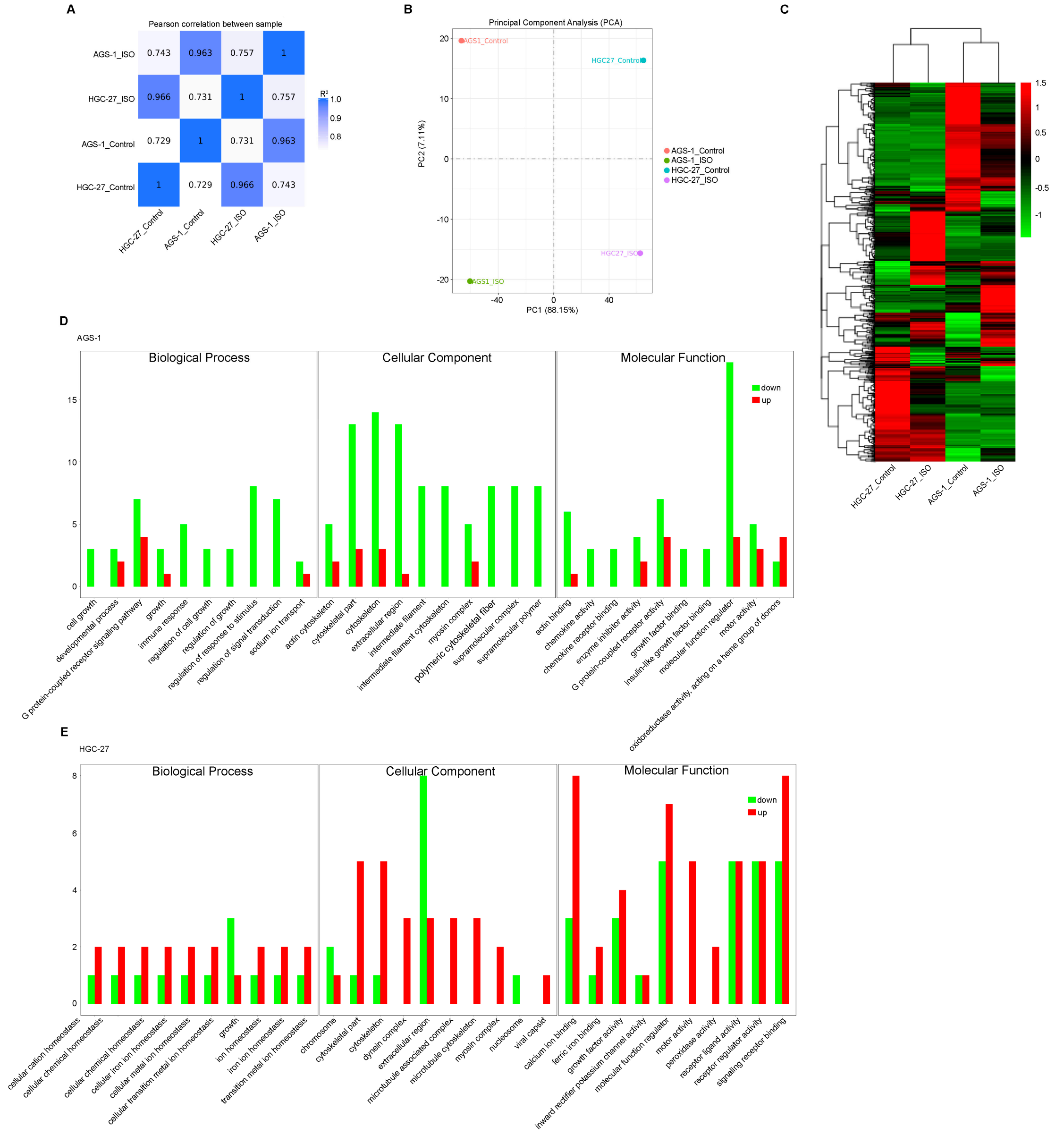

Figure S1. RNA-seq analysis of ISO-induced GC cells.

(A) The correlation plot of RNA-seq samples. **(B)** PCA analysis of RNA-seq samples. **(C)** DEGs of two types of GC cells revealed by Heatmap. **(D, E)** GO annotation of the DEGs in major three categories, including biological processes, cellular components and molecular functions. DEGs, differentially expressed genes.

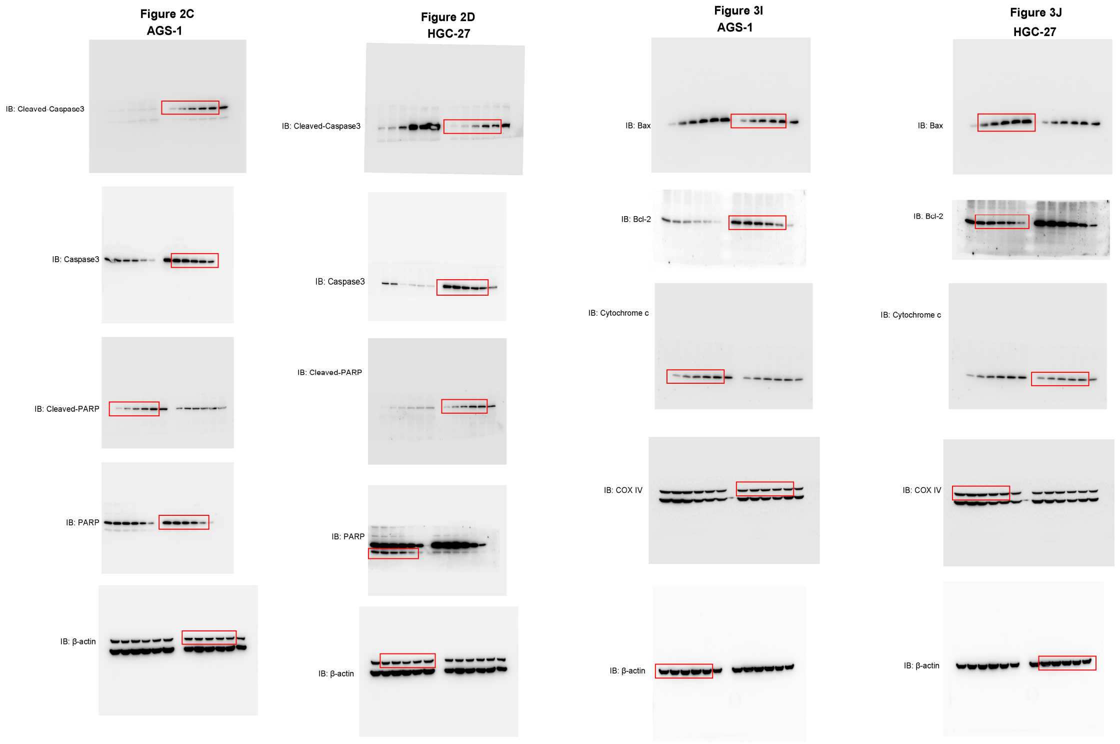

Figure S2. Images of the uncropped scans of immunoblots.

Boxes indicate the cropped sections used in the corresponding figures.
